# Spatiotemporal Epidemiology of Lumpy Skin Disease and Evaluation of the Heterologous Goatpox Vaccine: Insights into Immunogenicity and Impact

**DOI:** 10.3390/vaccines13060641

**Published:** 2025-06-13

**Authors:** Manjunatha Reddy Gundallahalli Bayyappa, Sai Mounica Pabbineedi, Sudeep Nagaraj, Shraddha Bijalwan, Sunil Tadakod, Chandana Ramesh Uma, Sanjay Pawar, Pathan Yahaya Khan, Vijay Kumar Teotia, Baldev Raj Gulati

**Affiliations:** 1 ICAR-National Institute of Veterinary Epidemiology and Disease Informatics (NIVEDI), Bengaluru 560064, India; mounica20sai@gmail.com (S.M.P.); sudeepcellculture@gmail.com (S.N.); shraddhabijalwan1998@gmail.com (S.B.); sunilnivedi@gmail.com (S.T.); chandanaru27999@gmail.com (C.R.U.); brgulati@gmail.com (B.R.G.); 2 Western Regional Disease Diagnostic Laboratory, State Level Animal Disease Investigation Laboratory, Aundh, Pune 411067, India; neovetsp@gmail.com (S.P.); dryapathan@gmail.com (P.Y.K.); 3 Department of Animal Husbandry and Dairying, Ministry of Fisheries, Animal Husbandry and Dairying, New Delhi 110001, India; roaqcs@gmail.com

**Keywords:** cell-mediated immune response, humoral immune response, immunogenicity, lumpy skin disease, spatial analysis, temporal analysis, vaccination

## Abstract

**Background:** Lumpy skin disease (LSD) is major transboundary disease affecting cattle and water buffaloes, indirectly causing huge socio-economic losses. Following its first outbreak in India in 2019, the heterologous Goatpox (Uttarkashi strain) vaccine mitigated LSD. **Objective:** Due to limited data on the spatiotemporal distribution of the disease, this study investigates its dynamics and presents findings from a field study conducted in Maharashtra, India. This study evaluates the safety, immunogenicity, and duration of immunity provided by a heterologous vaccine. Additionally, it examines post-vaccination responses in relation to factors such as age, gender, and breed. **Methods**: This study employed spatiotemporal analysis of lumpy skin disease (LSD) outbreaks from 2020 to 2024 using GeoDa (v1.22), incorporating Moran’s I and Getis-Ord Gi* statistics to identify spatial clustering patterns. A randomized field trial was conducted to evaluate vaccine safety and immunogenicity in 657 cattle across seven districts. Humoral immune responses were assessed using the serum neutralization test (SNT) and indirect enzyme-linked immunosorbent assay (ELISA), while cell-mediated immunity was evaluated via Interferon-gamma (IFN-γ) ELISA. For sero-monitoring, a total of 1925 serum samples from 22 districts were analyzed. Additionally, statistical analyses (*n* = 1925), including the Kappa Index, ANOVA, and logistic regression, were performed using SPSS v27 to investigate the influence of factors such as age, sex, and breed (significance level: *p* < 0.05). **Results:** LSD exhibited significant spatial clustering across Maharashtra. The Goatpox vaccine was 100% safe, with no adverse reactions. Protective antibody titers (≥1:8) were observed in 96.9% of vaccinated cattle by 14–21 days post-vaccination (dpv), peaking at 60 dpv before declining at 150 dpv. The cell-mediated immune response peaked at 28 dpv. Clinical monitoring for one year showed that only 2% of vaccinated cattle developed mild LSD symptoms after nine months, with no mortality. At six months post-vaccination, seroconversion was 69.7%, with breed significantly influencing seropositivity. **Conclusions:** This study confirms the Goatpox vaccine’s safety and strong immunogenicity in cattle, marking its first large-scale evaluation in the Indian subcontinent. Further research is needed to assess long-term immunity and protection against virulent LSD strains.

## 1. Introduction

Lumpy skin disease (LSD) is caused by the DNA virus classified under the family Poxviridae. The lumpy skin disease virus (LSDV), along with the Sheeppox virus (SPPV) and Goatpox virus (GTPV), belongs to the genus Capripoxvirus, which causes major economically significant diseases in domestic animals such as cattle, sheep, and goats, respectively [1]. Diseases caused by Capripoxviruses are generally host-specific and are confined to the African, European, and Asian subcontinents [1]. Initially identified in Zambia in 1929 and confined to the African subcontinent for several years, LSD has rapidly spread to Europe and the Middle East by 2012 [2]. According to the EFSA (2020) epidemiological report, LSD spread rapidly into eastern Asia in 2019, with first-time outbreaks reported in India, China, Bangladesh [3], and Myanmar [4] since 2019, which were followed by several outbreaks reported in the Asian subcontinent. It is indicated that both direct and indirect transmission routes are involved in spreading the disease [5]. LSDV can be transmitted directly through secretions from skin lesions as well as nasal, eye, and mouth discharges, which can contaminate the environment. Additionally, transmission through infected bull semen has also been identified as a potential route [2]. The virus can spread not only through direct contact but also indirectly via contaminated objects (fomites), feed, water, and mechanical vectors such as flies and ticks [3,6].

LSD primarily affects cattle, where *Bos taurus* breeds are more susceptible compared to the *Bos indicus* and it also affects water buffaloes [2]. Recently, the spillover of LSDV has been reported from in-contact animals such as Yak [7] and Mithun [8] in India. The disease is characterized by skin nodular lesions, with an incubation period of 1 to 4 weeks for natural infection [1]. LSD negatively impacts livestock by reducing milk yield, body weight, fertility, and growth rates, and can even lead to death. These effects result in substantial direct economic losses, along with indirect losses from trade limitations and treatment expenses [2]. Given its rapid cross-border transmission and severe impact on production, the World Organization for Animal Health (WOAH) has classified LSD as a significant notifiable disease [9]. In India, home to the largest livestock population globally [10], the disease has caused widespread outbreaks, with over 155,000 cattle deaths and more than 20 million animals affected [11,12]. This has translated into an estimated economic loss of USD 2217.26 million, highlighting the devastating impact of the disease on the dairy and cattle farming industries [13].

Given the rapid spread, economic burden, and zoonotic potential of LSDV, early detection and accurate diagnosis have become vital components of effective disease control strategies. Recent advancements in diagnostic techniques for LSDV have significantly improved the ability to detect and manage the disease effectively. Traditional diagnostic methods such as virus isolation, electron microscopy, immunofluorescence assays, and serological techniques like serum neutralization tests and ELISA have been widely used but often require specialized laboratories and trained personnel. To overcome these limitations, molecular diagnostics have emerged as powerful tools, with conventional and real-time PCR offering high sensitivity and specificity in identifying LSDV and distinguishing it from closely related Capripoxviruses such as Sheeppox virus and Goatpox virus. Furthermore, innovative isothermal amplification methods, including loop-mediated isothermal amplification (LAMP), recombinase polymerase amplification (RPA), and the Triple assay, have been developed to provide rapid, accurate, and field-deployable diagnostic solutions. These advancements support early detection and rapid response, which are critical for effective LSDV outbreak containment and control [14].

Complementing these diagnostic advancements, vaccination remains the primary strategy for the effective control and prevention of lumpy skin disease (LSD), especially in endemic and high-risk regions. Capripoxviruses share approximately 97% antigenic similarity, enabling cross-protection across species. This has led to the use of both homologous vaccines such as those based on the LSDV Neethling strain and heterologous vaccines derived from strains like Gorgan GTPV, Uttarkashi, Romanian SPPV, and others. While homologous vaccines generally offer robust protection [15], they are associated with some drawbacks, including the potential for virus shedding and the development of nodular skin lesions known as the “Neethling response.” These lesions may contain high viral loads and can contribute to further spread of the virus through insect vectors [16]. Live attenuated vaccines, especially those based on the Neethling strain, remain the most widely used due to their proven immunogenicity and durability. Nevertheless, concerns regarding mild adverse reactions, genetic stability, and the risk of recombination with wild strains have driven efforts to develop safer vaccine alternatives [17].

The LSDV vaccination with Capripoxvirus-based vaccines induces both cell-mediated and humoral immune responses. Cell-mediated immunity, particularly the activation of cytotoxic T lymphocytes (CTLs) and natural killer (NK) cells, plays a key role in clearing infected cells. A Th1-type response involving Interferon-gamma (IFN-γ) is essential for viral control and long-term protection [18]. In addition, the humoral immune response generates virus-specific antibodies, especially IgG, which help neutralize extracellular viruses and prevent reinfection. These antibodies, detectable by ELISA and virus neutralization tests, typically peak within 2–4 weeks post-vaccination and can persist for several months, offering sustained protection [19]. Together, these immune components are crucial for evaluating vaccine efficacy under field conditions.

India, which holds the highest bovine population (Cattle, Buffalo, Yak, and Mithun) with 302.79 million (20th livestock census, Department of Animal Husbandry and Dairying, 2019) in the world, reported its first LSD outbreak in the state of Odisha in 2019 [3,7]. Subsequently, the neighboring states were affected from 2020 to 2021, and by the end of 2022, the disease was reported throughout India, including the northwestern and southern states [8]. During this catastrophe caused by LSD in India, the state of Maharashtra also recorded its first case in 2020. After the distribution of LSD cases throughout the country, the Government of India (GoI) approved the emergency use of the commercially available Goatpox vaccine (Uttarkashi strain) to combat LSD in cattle [20]. However, no scientific experimental or field studies were conducted before the advising of heterologous vaccine use in India. The Maharashtra state reported the second-most deaths due to LSD (21). Hence, this study was carried out to understand the spatiotemporal distribution of LSD in Maharashtra, India, and to evaluate the safety, immunogenicity, and duration of immunity conferred by the heterologous Goatpox vaccine (Uttarkashi strain) under field conditions. In addition, post-vaccination sero-monitoring of the Goatpox vaccine against LSD in cattle was carried out to evaluate the vaccine’s effectiveness and risk factors such as age, breed, and gender influence on vaccine response.

## 2. Materials and Methods

### 2.1. Ethics Statement

The Animal Husbandry and Veterinary Services Department of Maharashtra permitted sample collection from cattle. Oral consent was also obtained from the respective animal owners by the appointed field veterinarians. This study was approved by the institutional animal ethics committee (No. NIVEDI/IAEC/2022/06 dated 23 July 2022).

### 2.2. Location, Study Design, Vaccine and Vaccination

A longitudinal and multiple sampling study was conducted in the state of Maharashtra to evaluate the safety, duration, and immunogenicity of heterologous vaccine. The study sites covered seven districts: Solapur, Satara, Kolhapur, Ratnagiri, Nagpur, Bandara, and Nashik. Under the seven districts named, 11 villages were randomly selected, each with six young and six adult cattle, with a total number of 132 seronegative animals enrolled. The animals were vaccinated with the live attenuated Goatpox (Ut-Uttarkashi strain) vaccine manufactured by [Hester Biosciences LtdMehsana, Gujarat, India., Batch number: A50013 manufactured on August 2022 with an expiry date of July 2024]. The single strain of Goatpox was permitted for vaccination under the Indian government-sponsored mass vaccination program.

The vaccine was administered subcutaneously to each cattle by veterinarians with a 1 mL dose containing 1 × 10^3^ tissue culture infectious dose50 (TCID_50_). The unvaccinated group were considered as the control (animals with antibody titer below 1:8) for the study in the field. However, these selected animals (control and vaccinated) were present in the villages with similar animal husbandry practices.

For the post-vaccination sero-monitoring, a simple stratified random sampling was followed. At six months post-vaccination, serum samples were obtained from cattle distributed across 22 districts. Each sample was processed for serological evaluation, and, concurrently, individual animal data, specifically sex, breed, and age, were recorded at the time of sampling, which was used for further analyses.

### 2.3. Sample Collection

The blood samples were collected aseptically from the jugular vein using BD Vacutainer tubes using standard protocol according to the World Organization for Animal Health guidelines for collection of samples. All the samples were collected and transported to the lab and centrifuged at 5000 revolutions per minute (rpm) for 30 min for sera separation. A total of 2582 serum samples were aliquoted and stored at −20 °C until further analysis.

### 2.4. Spatiotemporal Analysis

The spatiotemporal distribution in this study was performed using GeoDa software version 1.22. The information was gathered from the Department of Animal Husbandry, Maharashtra, where the LSD outbreaks were recorded. The Getis-Ord Gi* statistic was utilized to assess the spatial distribution of LSD cases reported across districts in Maharashtra between 2020 and 2024. This spatial clustering analysis was conducted at a 95% confidence interval to ensure reliable identification of areas exhibiting significant variations in disease prevalence. The spatial analysis technique was used to identify statistically significant clusters, distinguishing between higher (hotspots) or lower (cold spots) risk based on local LSD incidence relative to the surrounding districts of Maharashtra. Local indicators of spatial association (LISA) analyses were also performed for the LSD cases reported for the years 2020–2021, 2021–2022, 2022–2023, and 2023–2024 in Maharashtra, India. LISA clustering provided statistical validation for spatial patterns, ensuring that disease hotspots and cold spots are not just random occurrences but significant spatial phenomena. Moran’s I statistic used in this study provided a key measure that was used to evaluate the spatial autocorrelation, which assesses whether LSD cases in Maharashtra exhibit a clustered, dispersed, or random spatial distribution [21].

### 2.5. Indirect Enzyme-Linked Immunosorbent Assay (Indirect ELISA)

An in-house-developed indirect ELISA was used to detect Capripoxvirus-specific antibodies in 2,582 serum samples to assess humoral-mediated immunity (*n* = 657) and sero-monitoring (*n* = 1925). Briefly, LSDV recombinant antigen (A27L gene) was produced using field LSDV isolate (isolate name: Lumpy skin disease virus isolate LSDV/CHITRA-05/NIVEDI/ICAR/2020/India and accession number: OR863389) of NIVEDI expressed using prokaryotic expression system was coated at 50 ng per well, incubated, and then blocked. The assay was standardized and validated with known positive and negative panels. The serum samples (1:150 dilution) were added, followed by rabbit anti-bovine Immunoglobulin G Horseradish Peroxidase (IgG HRP) conjugate, and detection was performed using o-phenylenediamine dihydrochloride (OPD), with the reaction stopped with 1 M sulphuric acid (H_2_SO_4_). Optical density (OD) was measured at 492 nanometers (nm). Percentage positivity (PPV) was calculated, with values over 40% considered positive.

### 2.6. Serum Neutralization Test (SNT)

The serum neutralization test was performed for 657 serum samples to assess humoral-mediated immunity. Briefly, Madin–Darby Bovine Kidney (MDBK) cells were grown to 90% confluence. Serum samples were heat-inactivated and serially diluted. A viral suspension of 100 TCID^50^ was prepared from a field isolate (Accession number OR863389). Serum dilutions were incubated with LSDV and used to infect MDBK cells. Cytopathic effects (CPEs) were monitored, and a titer of 1:8 or more was considered positive.

### 2.7. Interferon-Gamma (IFN-γ) Assessment

The CMI response was assessed using a commercially available Bovine Interferon γ ELISA Kit (BT LAB, Shanghai, China) for 657 serum samples. Serum samples, Bovine IFN-γ antibody, and streptavidin-HRP were added to pre-coated plates, incubated, washed, and developed using substrate solutions. The reaction was stopped, and OD was measured at 450 nm using a microplate reader (Tecan Infinite F50, Mannedorf, Switzerland). IFN-γ is a general marker of cell-mediated immunity (CMI) and may reflect responses to a range of immunological stimuli, not just Capripoxvirus-specific responses.

### 2.8. Post-Vaccination Sero-Monitoring

To assess vaccine safety, animals were subjected to daily clinical monitoring for 28 days following vaccination. Observations included body temperature, nasal and ocular discharge, local reactions at the injection site, and the presence of nodular skin lesions, with evaluations conducted by field veterinarians [17]. Beyond the initial observation period, vaccinated animals were assessed weekly for up to one year using a standardized clinical scoring system to monitor overall health. Any suspected cases of LSD arising during the study were further investigated and confirmed through polymerase chain reaction (PCR) testing [8].

### 2.9. Data Analysis

Data was analyzed using the Statistical Package for the Social Sciences (IBM^®^ SPSS^®^ version 27). The Kappa index was used to evaluate the co-relationship between ELISA and SNT. One-way Analysis of Variance (ANOVA) and Dunnett’s post hoc test were used to assess antibody titer variation across different timepoints. Out of a total of 2745 records, 1925 data were selected for analysis based on the complete information for age, breed, and sex of the animals. The dataset was organized according to relevant factors and subsequently coded for use in SPSS version 27. In SPSS, each row in the Data View corresponded to an individual subject, while each column represented a variable such as age, breed, or gender. Within the Variable View, variables were appropriately defined with names, labels, data types, and assigned value labels. Then, logistic regression was used to determine the influence of different factors such as breed, age, and gender on seropositivity. Logistic regression was used to evaluate the odds ratio (OR) with a confidence interval (CI) of 95% to assess the strength of associations of factors, with statistical significance set at *p* < 0.05 [22].

## 3. Results

### 3.1. Spatiotemporal Epidemiology

**Figure 1:**
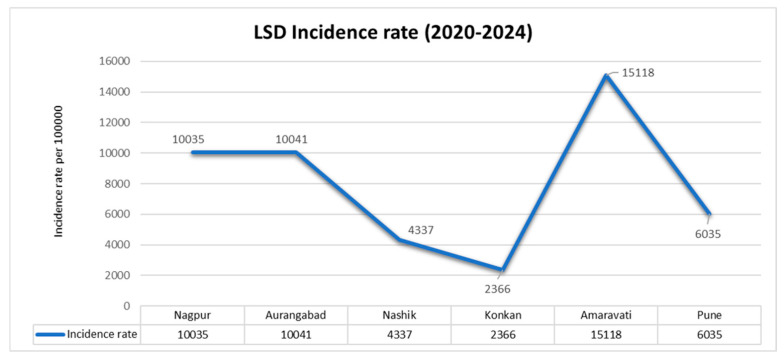
The incidence of LSD cases for every 100,000 cattle population through the years 2020–2024 in the divisions of Maharashtra.

The data collected from the Animal Husbandry Department of Maharashtra was analyzed for the epidemiological distribution of the disease across the various divisions of Maharashtra. The incidence rate of LSD was shown to be high in the Amaravati division, followed by the Aurangabad and Nagpur divisions. The lowest incidence rate was observed in the Konkan division, as shown in Figure 1.

**Figure 2:**
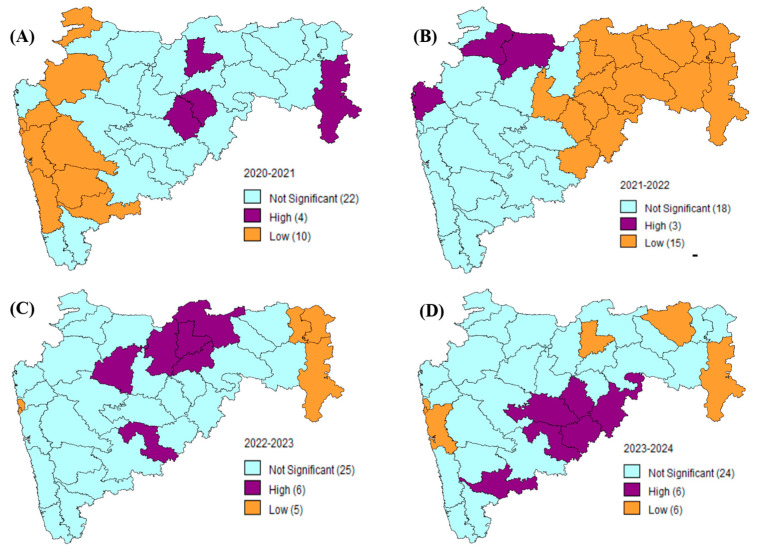
Getis-Ord-Gi* statistics showing the hotspots and cold spots for LSD cases in the state of Maharashtra for (**A**) 2020–2021, (**B**) 2021–2022, (**C**) 2022–2023, and (**D**) 2023–2024. Purple color indicates the hotspots with high LSD cases, orange color indicates the cold spots with low LSD cases, and the teal color indicates non-significant cases that were recorded across the state of Maharashtra.

The Getis-Ord Gi* statistics, as shown in Figure 2A–D, provide us with the crucial observations of the geospatial movement of the disease that could be aimed at disease management in the state through targeted strategies.

**Figure 3:**
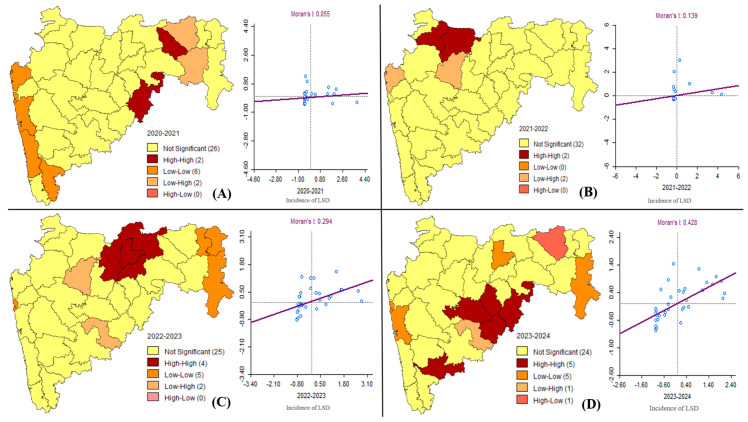
LISA clustering and Moran’s plot showing spatial distribution and outliners for LSD cases recorded from (**A**) 2020–2021, (**B**) 2021–2022, (**C**) 2022–2023, and (**D**) 2023–2024 in the state of Maharashtra, India. The high–high cluster (Brown color) implies a high number of cases in a district as well as the surrounding districts. Low–low cluster (orange color) indicates a smaller number of cases reported in a district and its surrounding districts. Low–high cluster (pastel orange color) implies low cases in the district but high numbers in the neighboring districts and vice versa in a high–low cluster (coral color).

Local indicators of spatial association (LISA) analysis, as shown in Figure 3, shows four categories of clusters displayed depending on the cases reported in each district. The correlation between the cases reported in each district (*x*-axis) and the number of cases reported in the neighboring districts (*y*-axis) are represented in the Moran’s I plots for the respective years, as mentioned earlier (Figure 3). For each year, the estimated Moran’s I value ranged at 0.055 (Z-score = 1.0004, *p* = 0.054) for 2020–2021, 0.1394 (Z-score = 2.1911, *p* = 0.04) for 2021–2022, 0.294 (Z-score = 2.8417, *p* = 0.004) for 2022–2023, and 0.4280 (Z-score = 4.192, *p* = 0.001) for 2023–2024. This indicates that the LSD cases form clear clustering, therefore indicating spatial distribution across the districts and neighboring districts.

### 3.2. Vaccine Safety Study

Throughout the study period, all the vaccinated animals remained healthy. No swelling was observed at the injection site, and all animals maintained normal body temperature, feed intake, and physiological norms.

**Figure 4:**
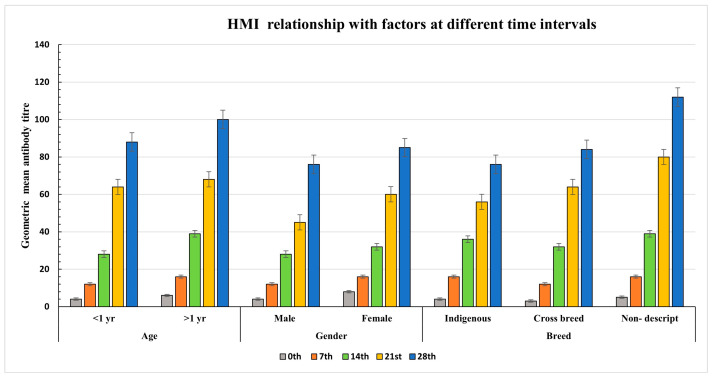
Humoral immune response (HMI) study at 0, 7, 14, 21 and 28 days post-vaccination, with animals administered with heterologous Goatpox vaccine against LSD in cattle, showing geometric mean antibody titers and its comparison with different factors such as age, gender, and breed.

Humoral immune response (HMI) was assessed in 657 serum samples collected on 0th, 7th, 14th, 21st, and 28th days post-vaccination (dpv), using both in-house indirect ELISA (iELISA) and the Serum Neutralization Test (SNT). Out of these, 551 samples were tested positive and 106 negatives by iELISA, while 549 were tested positive and 108 negatives by SNT, yielding a high degree of correlation between the two assays (Kappa index = 0.957) (Table 1 and Supplementary Table S1). At 0 dpv, animals exhibited low antibody titers (<1:8) (Supplementary Table S1), indicating the absence of prior immunity. By 7 dpv, 56% of animals had antibody titers above 1:8, and seroconversion significantly increased to 96.9% by 14 dpv, with titers peaking at 28 dpv. These changes in antibody titers were observed across all districts and are detailed in Table 1, while the overall titer trend is shown in Figure 4.

Further analysis indicated that immune responses varied by age, gender, and breed. Animals older than one year demonstrated significantly higher antibody titers compared to younger animals (Figure 4), and female cattle showed stronger responses than males (Figure 4). Breed-wise, non-descript animals exhibited the highest humoral responses, followed by crossbreeds and local breeds (Figure 4). A one-way ANOVA conducted for each district confirmed a statistically significant increase in antibody titers over time. Post hoc Dunnett’s T3 test revealed significant differences in titers between most time intervals, though no differences were observed between days 7 and 14 as well as 14 and 21 in Satara and Nagpur, or between days 21 and 28 in all districts (Supplementary Tables S2 and S3).

**Figure 5:**
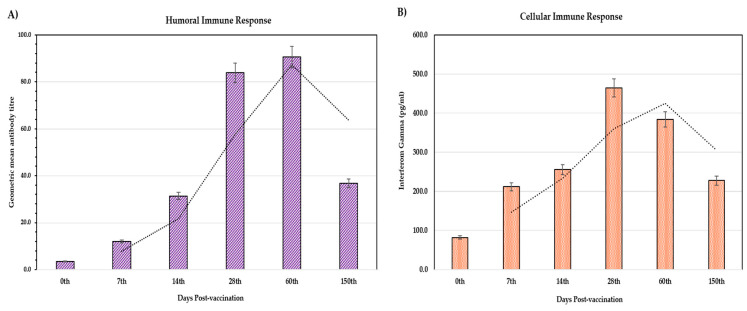
The humoral- and cell-mediated immune responses in the study animals vaccinated with heterologous Goatpox vaccine measured at 0, 7, 14, 28, 60, and 150 days post-vaccination. (**A**) The mean serum antibody levels appeared by day 7, peaked at 60 days post-vaccination, and declined at day 150. (**B**) The mean Interferon-gamma levels appeared by day 7, peaked at 28 days post-vaccination, and declined at day 150.

Cell-mediated immunity (CMI), evaluated through IFN-γ levels using a commercial ELISA, showed an early response by 7 dpv, peaking at 28 dpv before declining (Figure 5B). In contrast, the humoral response continued to rise, reaching its peak at 60 dpv and declining by 150 dpv (Figure 5A). Statistical analysis revealed significant differences in both IFN-γ and antibody titers across timepoints. Dunnett’s T3 post hoc test identified significant differences across most intervals, except between days 7 and 150, 14 and 150, as well as 28 and 60, indicating a plateau in immune response over these periods (Supplementary Tables S4 and S5).

### 3.3. Post-Vaccination Sero-Monitoring

Field effectiveness was evaluated by monitoring the vaccinated status for up to one year post-vaccination. Only two animals (one adult and one calf) developed clinical symptoms of LSD nine months post-vaccination. PCR analysis confirmed Capripoxvirus infection in both cases, indicating a low incidence of breakthrough infection and suggesting the strong protective efficacy of the vaccine. An assessment of vaccination coverage revealed that 9944 animals were vaccinated with the heterologous Goatpox vaccine across the selected and nearby districts (Akola, Pune, and Hingoli), while 206 animals remained unvaccinated, resulting in a high overall vaccination coverage of 97.1%.

A total of 1925 serum samples were collected from 22 districts across Maharashtra and analyzed six months after administration of the heterologous Goatpox vaccine. The overall seropositivity rate was found to be 69.7%. The distribution of seropositivity revealed notable regional differences. Indirect ELISA results showed the highest seropositivity rates in Osmanabad, Latur, Jalna, and Aurangabad, ranging from 82.07% to 83.6%. Districts such as Buldana, Akola, Amravati, and Yavatmal exhibited moderately high seropositivity levels (69.43% to 82.07%), whereas lower seropositivity rates (0% to 68.3%) were recorded in Ratnagiri, Thane, and Dhule. These findings suggest effective vaccine-induced immunity in certain regions, while others may require further evaluation or booster interventions due to suboptimal seroconversion. Univariate analysis indicated that breed had a statistically significant impact on antibody levels (*p* = 0.011). Although animals under two years of age showed an odds ratio greater than 1, the associated *p*-value was greater than 0.05, suggesting a non-significant trend. Multivariate logistic regression also confirmed a significant association between breed and seropositivity (*p* = 0.049), while the under-two age group had an OR value below 1, indicating reduced likelihood of seropositivity in this category (Supplementary Tables S6 and S7).

## 4. Discussion

The state of Maharashtra, being a significant cattle-producing region, has witnessed a surge in outbreaks since the initial detection of LSD in the country in 2019. As per the reports, the surrounding states of Maharashtra, such as Gujarat, Madhya Pradesh, Chhattisgarh, Telangana, and Karnataka, reported a high number of LSD cases in the year 2022 [23]. To mitigate the spread and impact of LSD, the Indian government authorized the use of a heterologous live attenuated Goatpox vaccine (Uttarkashi strain) [19,24]. This decision was based on antigenic similarities between LSDV, SPPV, and GTPV, which enable cross-protection [1]. Our randomized field study, conducted across districts in Maharashtra, demonstrated the safety of the vaccine, with no adverse post-vaccination reactions. These findings support earlier reports that heterologous Capripoxvirus vaccines do not cause significant side effects [25].

The analysis of LSD outbreaks in Maharashtra from 2020 to 2024 revealed distinct spatial clustering patterns, as reflected by Moran’s I values ranging from 0.055 to 0.428. The appearance of persistent high–high clusters following 2022 suggests a heightened risk of transmission and the establishment of localized high-risk zones. This pattern closely mirrors findings from a study by Bayir (2024) [21], which documented similar spatial trends in Turkey between 2013 and 2021. These findings suggest that animal movements, trade practices, and naive cattle populations likely contributed to the observed disease patterns consistent with East Africa and Turkey [21]. Many other risk factors also contribute to LSD outbreaks as described by Lee et al., 2024 [26].

The immunogenicity assessment in this study demonstrated that antibody levels began to rise as early as seven days post-vaccination, with a marked increase by day 14, indicating a strong humoral immune response. These findings are consistent with those of Varshovi et al. (2017) [27] and Tilahun et al. (2016) [28], who also reported a similar pattern of antibody development following vaccination. Additionally, the cell-mediated immune response, as indicated by Interferon-gamma (IFN-γ) levels, peaked around day 28 before gradually declining. This pattern is in agreement with observations by Norian et al. (2016) [29] and Milovanovic et al. (2019) [30], who identified IFN-γ as a key marker for post-vaccination CMI activity, supporting the notion of a typical immune trajectory following LSDV immunization.

Although a challenge trial was not performed, clinical monitoring over a 12-month period following vaccination showed that only 2% of vaccinated cattle exhibited symptoms of LSD. These breakthrough infections occurred approximately nine months post-vaccination, coinciding with significant regional outbreaks, which may indicate a decline in immunity over time. A similar observation was made by Gaber et al. (2022), who found that neutralizing antibody levels generated by heterologous vaccines begin to decrease approximately seven months after immunization [31]. Additionally, Osuagwuh et al. [32] reported protective immunity in the absence of detectable antibodies, suggesting that cell-mediated immunity (CMI) may play a critical role in ensuring long-term defense against LSDV.

Inter-district variations in antibody titers were observed, with districts such as Kolhapur and Ratnagiri exhibiting stronger immune responses, potentially reflecting differences in animal husbandry practices. Immune responses were also influenced by breed, age, and gender; animals older than one year and females demonstrated higher antibody titers. Crossbred and non-descript cattle showed greater seroconversion by day 28 post-vaccination. These findings align with previous studies reporting variability in vaccine-induced immunity influenced by genetic background and management practices [32]. These cases occurred in areas with ongoing outbreaks, suggesting high viral exposure pressure despite prior vaccination. The possible role of incomplete herd immunity in adjacent herds or villages could have facilitated transmission. This emphasizes the need for high vaccine coverage and continuous monitoring.

Serological monitoring conducted six months after vaccination, using an in-house indirect ELISA, showed a seropositivity rate of 74.6%, which falls slightly below the commonly accepted threshold of 80% required to achieve effective herd immunity with heterologous vaccines. This observation is consistent with findings reported by Kumar et al. (2023) [11]. In contrast, a study conducted in Armenia using a heterologous Sheeppox vaccine demonstrated even lower levels of immunity at both six- and eleven-months post-vaccination [33]. These findings highlight the importance of periodic serological monitoring to assess vaccine effectiveness and inform future vaccination strategies.

While vaccination is central to LSD control [34], other factors such as post-vaccination monitoring is equally essential to assess vaccine coverage and campaign effectiveness, as well as identify gaps in protection. Our study recorded an impressive 97.1% vaccination coverage across selected districts, suggesting successful implementation. However, due to annual mass booster campaigns, we were unable to evaluate vaccine-induced immunity beyond 12 months. Long-lasting immunity is essential to reduce the burden of revaccination, especially for smallholder and resource-poor farmers [33].

Therefore, selecting the most suitable vaccine strains has become increasingly important in light of emerging recombinant LSDV variants, which may arise from genetic recombination between vaccine and field strains potentially impacting virulence and reducing vaccine effectiveness [35]. This underscores the necessity for ongoing molecular monitoring and periodic reassessment of vaccination strategies under field conditions. Moreover, recent whole-genome sequencing efforts have revealed the circulation of diverse LSDV clades and recombinant strains across Eurasia and South Asia, further emphasizing the critical need for sustained genomic surveillance to track viral evolution and transmission dynamics [36]. In the Indian subcontinent, the genome sequencing studies suggested that the LSDV has evolved through geographic diversification, recombination events, and host adaptation, and the Indian LSD strains have shown rapid diversification and share common ancestors with strains from not only Kenya but also from China and Russia, indicating cross-border transmission. The divergence between field and vaccine strains emphasizes the need for combing through the genomic surveillance and vaccine updates to prevent the spread of LSDV [37].

In conclusion, this study provides novel and critical insights into both the epidemiology of LSD and the field-based safety, immunogenicity, duration of immunity given by the heterologous Goatpox vaccine. From the study, we note that areas with high LSD incidence in early years often experienced reduced cases in subsequent years due to good vaccination coverage. The limitation of the present study is lack of challenge to attribute the decreased number of LSD cases to vaccination with solely heterologous vaccines. However, this information is valuable for veterinary services to confirm that continued vaccination with good vaccine coverage is pivotal in the prevention and control of LSD along with other control measures. Detailed epidemiological studies involving modeling or systemic simulation approaches are needed for a better advanced outbreak response, which will help in prioritizing interventions in high-risk or naïve areas. Even though this is the first large-scale study in India assessing the sero-monitoring outcomes of a heterologous Capripoxvirus vaccine against LSDV in cattle under field conditions, there is need for long-term challenge studies and comparative vaccine field efficacy studies between heterologous and homologous vaccines in India.

## Figures and Tables

**Table 1:** The titer of seroconversion expressed in percentage in animals after vaccination with heterologous Goatpox vaccine (Uttarkashi strain) in the randomized field study across seven districts in the state of Maharashtra, India.

DPV	SNT Titre	Satara	Solapur	Kolhapur	Ratnagiri	Nashik	Bhandra	Nagpur
7th day	1:4	16.70	00.00	00.00	04.16	08.30	12.50	00.00
	1:8	33.33	33.33	37.50	29.20	45.80	37.50	50.00
	1:16	50.00	58.30	45.80	41.70	37.50	45.80	25.00
	1:32	00.00	08.30	16.70	25.00	08.30	04.16	25.00
14th day	1:8	08.30	00.00	00.00	00.00	00.00	00.00	00.00
	1:16	50.00	00.00	25.00	08.30	04.16	08.30	50.00
	1:32	33.33	66.67	33.30	29.20	37.50	37.50	25.00
	1:64	08.30	33.33	33.30	45.80	45.80	37.50	25.00
	1:128	00.00	00.00	08.30	16.70	12.50	16.70	00.00
21st day	1:16	08.30	00.00	00.00	00.00	04.16	8.30	00.00
	1:32	41.70	00.00	00.00	00.00	37.50	45.80	50.00
	1:64	41.70	83.33	62.50	29.20	50.00	41.70	41.70
	1:128	8.30	16.67	16.70	33.30	04.16	4.160	08.30
	>1:128	00.00	00.00	20.80	37.50	00.00	00.00	00.00
28th day	1:32	22.20	00.00	00.00	00.00	08.30	16.70	00.00
	1:64	66.66	58.30	20.80	04.16	75.00	79.20	91.60
	1:128	11.11	41.70	41.70	29.20	16.70	04.16	08.30
	>1:128	00.00	00.00	37.50	66.70	00.00	00.00	00.00

DPV = days post-vaccination; SNT = serum neutralization test.

## Data Availability

The data presented in this study are available on request from the corresponding author. The data are not publicly available due to the laid-down rules of the institute.

## Supplementary Materials

The following supporting information can be downloaded at: https://www.mdpi.com/article/10.3390/vaccines13060641/s1; Table S1: The rate of seroconversion in the study animals before and after vaccination with heterologous Goatpox vaccine (Uttarkashi strain) in the randomized field study across seven districts in the state of Maharashtra, India; Table S2: Analysis of Variance (ANOVA) of antibody titers obtained by serum neutralization among the vaccinated animals in various districts of Maharashtra at different time intervals post-vaccination; Table S3: Multiple comparisons using Dunnete T3 of antibody titers obtained by serum neutralization among the vaccinated animals in various districts of Maharashtra at different time intervals post-vaccination; Table S4: Analysis of Variance (ANOVA) among the vaccinated animals for cell-mediated and humoral-mediated immunity at different time intervals post-vaccination; Table S5: Multiple comparisons using Dunnete T3 among the vaccinated animals for cell-mediated and humoral-mediated immunity at different time intervals post-vaccination; Table S6: Univariate analysis of age, gender, breed, and state on the seropositivity after six months post-vaccination with heterologous Goatpox vaccine against LSD in cattle; Table S7: Multivariate logistic regression analysis of age, gender, breed, and state on seropositivity after six months post-vaccination with heterologous Goatpox vaccine against LSD in cattle.
