# Supplementary material for: Spatiotemporal Epidemiology of Lumpy Skin Disease and Evaluation of the Heterologous Goatpox Vaccine: Insights into Immunogenicity and Impact
Source: Vaccines (Basel). 2025 Jun 13;13(6):641. doi: 10.3390/vaccines13060641 (PMC12197790; doi:10.3390/vaccines13060641)
Supplement: Supplementary file 1 [file vaccines-13-00641-s001.zip › vaccines-3601523-supplementary.pdf]

Supplementary tables

Supplementary table 1. The rate of seroconversion in the study animals before and after vaccination with heterologous Goatpox vaccine (Uttarkashi strain) in the randomized field study across seven districts in the state of Maharashtra, India.

District	SNT titre	0	7	14	21	28
Satara	0	33.33% (4/12)	-	-	-	-
	1:2	41.7% (5/12)	-	-	-	-
	1:4	25% (3/12)	16.7% (2/12)	-	-	-
	1:8	-	33.33% (4/12)	8.3% (1/12)	-	-
	1:16	-	50% (6/12)	50% (6/12)	8.3% (1/12)	-
	1:32	-	-	33.33% (4/12)	41.7% (5/12)	22.2% (2/9)
	1:64	-	-	8.3% (1/12)	41.7% (5/12)	66.66 % (6/9)
	1:128	-	-	-	8.3% (1/12)	11.11% (1/9)
Solapur	0	41.7% (5/12)	-	-	-	-
	1:2	58.3% (7/12)	-	-	-	-
	1:4	-	-	-	-	-
	1:8	-	33.33% (4/12)	-	-	-
	1:16	-	58.3% (7/12)	-	-	-
	1:32	-	8.3% (1/12)	66.67% (8/12)	-	-
	1:64	-	-	33.33% (4/12)	83.33% (10/12)	58.3% (7/12)
	1:128	-	-	-	16.67% (2/12)	41.7% (5/12)
Kolhapur	0	33.3% (8/24)	-	-	-	-
	1:2	45.8% (11/24)	-	-	-	-
	1:4	20.8% (5/24)	-	-	-	-
	1:8	-	37.5% (9/24)	-	-	-
	1:16	-	45.8% (11/24)	25% (6/24)	-	-
	1:32	-	16.7% (4/24)	33.3% (8/24)	-	-
	1:64	-	-	33.3% (8/24)	62.5% (15/24)	20.8% (5/24)
	1:128	-	-	8.3% (2/24)	16.7% (4/24)	41.7% (10/24)
	>1:128	-	-	-	20.8% (5/24)	37.5% (9/24)
Ratnagiri	0	12.5% (3/24)	-	-	-	-
	1:2	33.3% (8/24)	-	-	-	-
	1:4	54.2% (13/24)	4.16% (1/24)	-	-	-
	1:8	-	29.2% (7/24)	-	-	-
	1:16	-	41.7% (10/24)	8.3% (2/24)	-	-
	1:32	-	25% (6/24)	29.2% (7/24)	-	-
	1:64	-	-	45.8% (11/24)	29.2% (7/24)	4.16% (1/24)
	1:128	-	-	16.7% (4/24)	33.3% (8/24)	29.2% (7/24)
	>1:128	-	-	-	37.5% (9/24)	66.7% (16/24)
Nashik	0	20.8% (5/24)	-	-	-	-
	1:2	58.3% (14/24)	-	-	-	-

	1:4	20.8% (5/24)	8.3% (2/24)	-	-	-
	1:8	-	45.8% (11/24)	4.16% (1/24)	-	-
	1:16	-	37.5% (9/24)	37.5% (9/24)	4.16% (1/24)	
	1:32	-	8.3% (2/24)	45.8% (11/24)	37.5% (9/24)	8.3% (2/24)
	1:64	-	-	12.5% (3/24)	50% (12/24)	75% (18/24)
	1:128	-	-	-	4.16% (1/24)	16.7% (4/24)
**Bhandara**	0	33.3% (8/24)	-	-	-	-
	1:2	45.8% (11/24)	-	-	-	-
	1:4	20.8% (5/24)	12.5% (3/24)	-	-	-
	1:8	-	37.5% (9/24)	8.3% (2/24)	-	-
	1:16	-	45.8% (11/24)	37.5% (9/24)	8.3% (2/24)	-
	1:32	-	4.16% (1/24)	37.5% (9/24)	45.8% (11/24)	16.7% (4/24)
	1:64	-	-	16.7% (4/24)	41.7% (10/24)	79.2% (19/24)
	1:128	-	-	-	4.16% (1/24)	4.16% (1/24)
**Nagpur**	0	25% (3/12)	-	-	-	-
	1:2	50% (6/12)	-	-	-	-
	1:4	25% (3/12)	-	-	-	-
	1:8	-	50% (6/12)	-	-	-
	1:16	-	41.7% (5/12)	50% (6/12)	-	-
	1:32	-	8.3% (1/12)	25% (3/12)	50% (6/12)	-
	1:64	-	-	25% (3/12)	41.7% (5/12)	91.6% (11/12)
	1:128	-	-	-	8.3% (1/12)	8.3% (1/12)

Supplementary Table 2. Analysis of Variance (ANOVA) of antibody titers obtained by serum neutralization among the vaccinated animals in various districts of Maharashtra at different time intervals post-vaccination.

District	Parameters	Sum of Squares	Mean Square	Frequency	Significance
Solapur	Between Groups	67694.361	16923.59	42.302	0
	Within Groups	22003.865	400.07	--	--
	Total	89698.226	--	--	--
Satara	Between Groups	22741.47	5685.367	12.449	0
	Within Groups	18267.08	456.677		
	Total	41008.55	--	--	--
Kolhapur	Between Groups	289317	72329.26	37.539	0
	Within Groups	154141	1926.763	--	--
	Total	443458.1	--	--	--
Ratnagiri	Between Groups	272995.1	68248.76	35.729	0
	Within Groups	95509.14	1910.183	--	--
	Total	368504.2	--	--	--
Nagpur	Between Groups	27901.21	6975.303	18.981	0
	Within Groups	14699.42	367.486	--	--
	Total	42600.64	--	--	--
Bandara	Between Groups	42435.27	10608.82	39.528	0
	Within Groups	24154.81	268.387	--	--
	Total	66590.08	--	--	--
Nashik	Between Groups	53379.78	13344.94	47.083	0

Within Groups	25509.27	283.436	--	--
Total	78889.05	--	--	--

Supplementary Table 3. Multiple Comparisons using Dunnete T3 of antibody titers obtained by serum neutralization among the vaccinated animals in various districts of Maharashtra at different time intervals post-vaccination.

District	Time groups	Mean difference	Standard Error	Significance	95% Confidence Interval (Lower limit and upper limit)	
Nagpur	0th -7th	-11.49917*	1.9505	0.001	-18.0723	-4.926
	0th-14th	-39.49837*	4.55893	0	-54.9734	-24.0234
	0th-21st	-71.50181*	7.19695	0	-95.9556	-47.048
	0th-28th	-87.50034*	9.51719	0	-119.847	-55.1536
	7th-14th	-27.99920*	4.9408	0	-43.9712	-12.0272
	7th-21st	-60.00264*	7.44471	0	-84.7027	-35.3026
	7th-28th	-76.00117*	9.7059	0	-108.516	-43.4866
	14th-21st	-32.00344*	8.509	0.013	-58.7164	-5.2905
	14th-28th	-48.00197*	10.54437	0.003	-81.7913	-14.2126
	21st-28th	-15.99853	11.9246	0.847	-53.0389	21.0418
Satara	0th -7th	-9.33268	2.96024	0.098	-20.0862	1.4208
	0th-14th	-22.66532*	5.63297	0.03	-43.2393	-2.0913
	0th-21st	-44.88695*	11.31938	0.033	-86.2954	-3.4785
	0th-28th	-60.88957*	9.24436	0.001	-94.6984	-27.0807
	7th-14th	-13.3326	6.344	0.381	-34.5317	7.8664
	7th-21st	-35.5543	11.6895	0.106	-76.9891	5.8806
	7th-28th	-51.55689*	9.69403	0.003	-85.4711	-17.6426
	14th-21st	-22.2216	12.63376	0.586	-64.6611	20.2178
	14th-28th	-38.22425*	10.81395	0.032	-73.8075	-2.641
	21st-28th	-16.0026	14.60615	0.944	-62.9724	30.9672
Kolhapur	0th -7th	-9.41122*	1.0128	0	-12.6053	-6.2171
	0th-14th	-39.99882*	6.9684	0	-62.2805	-17.7171
	0th-21st	-98.35120*	15.579	0	-148.178	-48.5243
	0th-28th	-154.81503*	16.57036	0	-207.813	-101.817
	7th-14th	-30.58760*	7.0335	0.004	-52.969	-8.2062
	7th-21st	-88.93998*	15.60823	0	-138.811	-39.0694
	7th-28th	-145.40381*	16.59784	0	-198.443	-92.3648
	14th-21st	-58.35238*	17.0631	0.023	-110.937	-5.7677
	14th-28th	-114.81621*	17.97279	0	-170.371	-59.2614
	21st-28th	-56.4638	22.74132	0.161	-124.515	11.5871
Ratnagiri	0th -7th	-11.45348*	2.87207	0.021	-21.3712	-1.5357
	0th-14th	-37.27176*	5.86194	0.001	-57.5773	-16.9662
	0th-21st	-107.08854*	17.45454	0.001	-167.604	-46.5733
	0th-28th	-188.53157*	22.83048	0	-267.689	-109.374
	7th-14th	-25.81828*	6.51555	0.012	-46.9477	-4.6888
	7th-21st	-95.63506*	17.68477	0.002	-156.286	-34.9842
	7th-28th	-177.07810*	23.00697	0	-256.331	-97.825
	14th-21st	-69.81678*	18.40828	0.022	-131.192	-8.4417
	14th-28th	-151.25981*	23.56766	0	-230.963	-71.5572
	21st-28th	-81.443	28.73557	0.094	-171.586	8.7003
Nagpur	0th -7th	-9.11046	2.72351	0.075	-18.9994	0.7785
	0th-14th	-21.55437*	5.41743	0.032	-41.3422	-1.7665
	0th-21st	-46.44212*	10.81926	0.021	-86.0213	-6.8629

	0th-28th	-67.77987*	7.11827	0	-93.8026	-41.7572
	7th-14th	-12.4439	6.04514	0.406	-32.7383	7.8504
	7th-21st	-37.3317	11.14682	0.068	-76.9259	2.2626
	7th-28th	-58.66941*	7.6069	0	-84.8992	-32.4397
	14th-21st	-24.8877	12.0906	0.406	-65.4765	15.701
	14th-28th	-46.22550*	8.93286	0.001	-75.0726	-17.3784
	21st-28th	-21.3378	12.94232	0.655	-63.6164	20.9409
Bandara	0th -7th	-7.99956*	1.56426	0.001	-12.9036	-3.0955
	0th-14th	-20.41937*	3.0354	0	-29.9714	-10.8674
	0th-21st	-40.62984*	5.97921	0	-59.4651	-21.7946
	0th-28th	-57.47477*	4.8352	0	-72.7033	-42.2462
	7th-14th	-12.41980*	3.39884	0.011	-22.7203	-2.1193
	7th-21st	-32.63027*	6.17167	0	-51.8081	-13.4524
	7th-28th	-49.47521*	5.07126	0	-65.1383	-33.8121
	14th-21st	-20.21047	6.69748	0.052	-40.516	0.0951
	14th-28th	-37.05541*	5.69951	0	-54.1722	-19.9386
	21st-28th	-16.84494	7.68256	0.285	-39.7183	6.0284
Nashik	0th -7th	-7.47321*	1.50776	0.001	-12.2035	-2.7429
	0th-14th	-21.36645*	2.93456	0	-30.603	-12.1299
	0th-21st	-44.52504*	5.87197	0	-63.0236	-26.0265
	0th-28th	-63.89613*	5.41783	0	-80.9631	-46.8291
	7th-14th	-13.89324*	3.28615	0.002	-23.853	-3.9335
	7th-21st	-37.05183*	6.05534	0	-55.876	-18.2276
	7th-28th	-56.42293*	5.61604	0	-73.8459	-39
	14th-21st	-23.15858*	6.55785	0.015	-43.0547	-3.2624
	14th-28th	-42.52968*	6.15453	0	-61.1337	-23.9257
	21st-28th	-19.3711	7.98415	0.178	-43.0897	4.3475

* The mean difference is significant at the 0.05 level.

Supplementary Table 4. Analysis of variance (ANOVA) among the vaccinated animals for cell-mediated and humoral-mediated immunity at different time intervals post-vaccination.

Type of immune response	Parameters	Sum of Squares	Degrees of freedom	Mean Square	Frequency	Significance
Cell mediated immune response (CMI)	Between Groups	2810890.992	5	562178.198	59.74	0
	Within Groups	1166884.908	124	9410.362	--	--
	Total	3977775.899	129	--	--	--
Humoral mediated immune response (HMI)	Between Groups	233311.234	5	46662.25	34.96	0
	Within Groups	204214.2	153	1334.733		
	Total	437525.434	158	--	--	--

Supplementary Table 5. Multiple Comparisons using Dunnete T3 among the vaccinated animals for cell-mediated and humoral-mediated immunity at different time intervals post-vaccination.

Type of immune response	Time groups	Mean difference	Standard Error	Significance	95% Confidence Interval (Lower limit and upper limit)	
Cell mediated immune response (CMI)	0th -7th	-129.69637*	10.16005	0	-161.032	-98.3607
	0th-14th	-173.62583*	12.59461	0	-211.723	-135.528
	0th-28th	-382.64917*	28.1487	0	-469.584	-295.714
	0th-60th	-301.92194*	28.81764	0	-405.73	-198.114

	0th -150th	-145.56194*	10.03568	0	-179.242	-111.882
	7th-14th	-43.92947*	10.07738	0.001	-75.0117	-12.8473
	7th-28th	-252.95280*	27.11602	0	-337.379	-168.527
	7th-60th	-172.22558*	27.80982	0.002	-275.563	-68.8879
	7th -150th	-15.86558	6.60638	0.428	-50.2396	18.5085
	14th-28th	-209.02333*	28.11896	0	-295.884	-122.163
	14th-60th	-128.29611*	28.7886	0.013	-232.083	-24.5092
	14th-150th	28.06389	9.95197	0.145	-5.4315	61.5592
	28th-60th	80.72722	38.24276	0.446	-40.845	202.2994
	28th-150th	237.08722*	27.06966	0	152.6392	321.5353
	60th-150th	156.36000*	27.76462	0.003	52.8873	259.8327
Humoral mediated immune response (HMI)	0th -7th	-9.83333*	1.35521	0	-14.0215	-5.6452
	0th-14th	-34.72222*	4.25496	0	-48.0038	-21.4407
	0th-28th	-96.50000*	11.5631	0	-132.632	-60.3678
	0th-60th	-92.05556*	10.67484	0	-132.273	-51.8384
	0th -150th	-34.45556*	6.41362	0.043	-67.4299	-1.4812
	7th-14th	-24.88889*	4.42631	0	-38.583	-11.1948
	7th-28th	-86.66667*	11.62725	0	-122.948	-50.3854
	7th-60th	-82.22222*	10.74429	0	-122.443	-42.0019
	7th -150th	-24.62222	6.52856	0.125	-56.9141	7.6697
	14th-28th	-61.77778*	12.30695	0	-99.7201	-23.8354
	14th-60th	-57.33333*	11.47641	0.004	-97.9752	-16.6915
	14th-150th	0.26667	7.67399	1	-29.4955	30.0288
	28th-60th	4.44444	15.72605	1	-45.0738	53.9627
	28th-150th	62.04444*	13.2095	0.001	20.5774	103.5115
	60th-150th	57.60000*	12.43936	0.007	14.1296	101.0704

* The mean difference is significant at the 0.05 level.

Supplementary Table 6. Univariate analysis of age, gender, breed and state on the seropositivity after six months post-vaccination with heterologous Goatpox vaccine against LSD in cattle.

**Risk factors**		**Odds Ratio**	**95% Confidence Interval (CI)**	**p-value**
**Gender**	Male	0.727	0.493-1.072	0.108
	Female	1	-	-
**Breed**	Crossbreeds	1.386	1.077-1.782	0.011
	Local breeds	1	-	-
**Age**			0.398	
	<2 years	1.167	0.721-1.887	0.53
	2-4 years	0.865	0.545-1.374	0.539
	4-8 years	0.938	0.614-1.432	0.766
	>8 years	1	-	-

Supplementary Table 7. Multivariate logistic regression analysis of age, gender, breed and state on the seropositivity after six months post-vaccination with heterologous Goatpox vaccine against LSD in cattle.

**Risk factors**		**Odds Ratio (OR)**	**95% Confidence Interval (CI)**	**p-value**
**Gender**	Male	0.907	0.585-1.407	0.664
	Female	1	-	-
Breed	Crossbreeds	1.33	1.002-1.766	0.049
	Local breeds	1	-	-

Age	0.818			
	<2 years	0.805	0.653-1.732	1.063
	2-4yrs	0.869	0.601-1.538	0.961
	4-8 yrs	0.684	0.709-1.691	1.095
	>8yrs	1	-	-
